# Application of metabolomics in the diagnosis of non-alcoholic fatty liver disease and the treatment of traditional Chinese medicine

**DOI:** 10.3389/fphar.2022.971561

**Published:** 2022-08-25

**Authors:** Mingmei Shao, Yifei Lu, Hongjiao Xiang, Junmin Wang, Guang Ji, Tao Wu

**Affiliations:** ^1^ Baoshan District Hospital of Intergrated Traditional Chinese and Western Medicine, Shanghai, China; ^2^ Institute of Interdisciplinary Integrative Medicine Research, Shanghai University of Traditional Chinese Medicine, Shanghai, China; ^3^ Institute of Digestive Disease, Longhua Hospital, Shanghai University of Traditional Chinese Medicine, Shanghai, China

**Keywords:** Non-alcoholic fatty liver disease, metabolomics, metabolites, biomarkers, traditional Chinese medicine, research progress

## Abstract

Non-alcoholic fatty liver disease (NAFLD) is the most common chronic liver disease around the world, and it often coexists with insulin resistance-related diseases including obesity, diabetes, hyperlipidemia, and hypertension, which seriously threatens human health. Better prevention and treatment strategies are required to improve the impact of NAFLD. Although needle biopsy is an effective tool for diagnosing NAFLD, this method is invasive and difficult to perform. Therefore, it is very important to develop more efficient approaches for the early diagnosis of NAFLD. Traditional Chinese medicine (TCM) can play a certain role in improving symptoms and protecting target organs, and its mechanism of action needs to be further studied. Metabolomics, the study of all metabolites that is thought to be most closely associated with the patients’ characters, can provide useful clinically biomarkers that can be applied to NAFLD and may open up new methods for diagnosis. Metabolomics technology is consistent with the overall concept of TCM, and it can also be used as a potential mechanism to explain the effects of TCM by measuring biomarkers by metabolomics. Based on PubMed/MEDLINE and other databases, this paper retrieved relevant literature NAFLD and TCM intervention in NAFLD using metabolomics technology in the past 5 years were searched, and the specific metabolites associated with the development of NAFLD and the potential mechanism of Chinese medicine on improving symptoms were summarized.

## Introduction

Non-alcoholic fatty liver disease (NAFLD) is a metabolic stress-associated liver damage closely linked to insulin resistance and genetic predisposition. It is a clinicopathological syndrome characterized by diffuse steatosis. These include nonalcoholic simple fatty liver (NAFL), non-alcoholic steatohepatitis (NASH) and liver cirrhosis (Rowell and Anstee, 2015). Epidemiological reports indicate that the global prevalence of NAFLD has reached 25%, and there is a trend of younger patients with the incidence rate increasing year by year (Younossi et al., 2018; Cotter and Rinella, 2020; Powell et al., 2021). The global burden of end-stage liver disease is expected to increase 2–3 times by 2030 (Sanyal, 2019). With changes in people’s lifestyles, the prevalence of NAFLD in the Chinese population is also increasing year by year (Zhu et al., 2015; Xiao et al., 2019; Wong and Chan, 2021). By 2030, the total NAFLD-affected population in China is projected to up to 314 million cases, which is the country with the largest increase in NAFLD prevalence in the world (Zhou et al., 2020; Wong and Chan, 2021). Even if liver needle biopsy is the most definitive criteria for the diagnosis of NAFLD, this method is invasive and difficult to perform. Despite its incidence and significance of early diagnosis, there is short of valuable potential biomarkers for NAFLD/NASH diagnosis and the monitoring of the effects of related treatments. There is a crucial need to strengthen the development of clinical biomarkers for NAFLD to improve support for non-invasive diagnosis.

Metabolomics aims to quantitatively describe the dynamic multi-parameter changes of metabolites in organisms, and discovers the association between metabolite fluctuations and physiological and pathological alterations (Beger et al., 2016; Muthubharathi et al., 2021). Lipidomics, a branch of metabolomics, is to identify the key lipid biomarkers by comparing the changes of all lipid small molecule metabolites in the body under different pathophysiological states, and to investigate the mechanism of lipid in the physiological and pathological changes of the body (Zhao et al., 2014; Yang and Han, 2016). Metabolomics includes untargeted and targeted metabolomics analysis. Untargeted metabolomics mainly focuses on the global detection and relative quantification of small molecules in a sample, whereas targeted metabolomics mainly focuses on measuring well-defined metabolomes with absolute quantification (Johnson et al., 2016; Schrimpe-Rutledge et al., 2016). A large number of metabolomics and lipidomics experimental studies have demonstrated that the metabolic pathways in NAFLD/NASH have changed, mainly involving alterations in several aspects of amino acid, bile acid and lipid metabolism, including circulating fatty acids, branched-chain amino acids (BCAAs), phospholipids, triglycerides (TGs), and secondary bile acids these pathways (Caussy et al., 2019b; Masoodi et al., 2021; Nimer et al., 2021). Compared to traditional biomarkers, metabolomics can more accurately reflect the current metabolic state of the body, allowing us to profile patients’ disease progression by potential biomarker, not just general parameters (Rello et al., 2018). We have reviewed metabolomics studies in NAFLD and NASH in recent 5 years. Metabolomics studies have identified a variety of possible NAFLD-related biomarkers, which are significant for the early diagnosis and pathogenesis of NAFLD, although the clinical universality of these biomarkers remains to be further verified.

There are no clinically approved drugs for the treatment of NAFLD. Traditional Chinese medicine (TCM) is becoming more and more popular all over the world, especially in early intervention, combined treatment, and personalized medicine (Xiao et al., 2017; Qiu et al., 2020; Yan et al., 2020). It shows great advantages. Modern studies have shown that TCM can effectively regulate the body’s lipid metabolism (Yang et al., 2020; Gong X. et al., 2021), delay the process of NAFLD, and provide a therapeutic method for treating NAFLD. Metabolomics quantitatively analyzes metabolites in organisms to find biomarkers for early prediction of disease, which is highly consistent with the overall thinking of TCM. Therefore, the application of metabolomics technology is useful to find the mechanism of TCM in the therapy of NAFLD. The research is of great significance and bridges the gap between TCM and modern research.

## Metabolomics applied to the diagnosis of NAFLD

### Metabolomics characterization of NAFLD in clinical studies

In recent years, metabolomics technology has become more and more widely used in clinical research, accounting for 95% of the global clinical trial workload (Jacob et al., 2019). Using metabolomics technology to understand the changes of metabolites and metabolic pathways before and after the onset of the disease, to further understand the pathogenesis of the disease, it is possible to predict the progression of the disease, early diagnosis and improve the level of treatment (Ishikawa et al., 2016; LeWitt et al., 2017; Peña-Bautista et al., 2019; Liu X. et al., 2020; Amerikanou et al., 2021; Liu S. et al., 2021). Goffredo et al. measured a total of 180 plasma metabolites in 78 obese adolescents by a targeted metabolomics approach and found that obese NAFLD adolescents had higher plasma levels of isoleucine, valine, lysine and tryptophan. Leucine and valine are BCAAs which are inversely related to peripheral and hepatic insulin sensitivity. This study pointed out that the early changes of BCAAs metabolism may be an important signal for the advancement and progress of obese adolescents with NAFLD, and the BCAAs metabolic pathway may also be a potential goal for developing drugs to treat obese adolescents with NAFLD (Goffredo et al., 2017).

Jin et al. (2016) used a metabolomics approach to analyze 9,583 metabolites in 39 obese adolescents with NAFLD. Of these, 478 metabolites were related to the presence of NAFLD compared to obese adolescents without NAFLD. Pathway analysis demonstrated that several major amino acid pathways in NAFLD were dysregulated, including tyrosine metabolism, tryptophan metabolism, BCAA metabolism, glycine metabolism, serine and other amino acid metabolisms, of which tyrosine metabolism was the most affected. The tyrosine metabolic pathway may be an important pathway in the progression of NAFLD in children and deserves further study.

Hu et al. (2021) used ultra-performance liquid chromatography-orbitrap mass spectrometry (UPLC-Orbitrap MS) to study the metabolites in serum of 112 NAFLD patients, and found 55 differential metabolites (with variable importance in projection (VIP) > 1 and *p*  < 0.01), including the area under the curve (AUC) of 15 metabolites including phenylalanine, oleic acid and tryptophan were all above 0.9, indicating that these serum metabolites have high diagnostic value in NAFLD. These serum metabolites can serve as the basis for clinical NAFLD screening.

Barr et al. (2010) used UPLC-MS to study the serum metabolites of NAFLD patients and found that the contents of phosphatidylcholine (PC), lysophosphatidylcholine (LPC), sphingomyelin, free fatty acids, bile acids and organic acids, were increased in NAFLD patients, and it is proposed that a new direction to study the progress of NAFLD may start from the changes of these substances.

A metabolomics study of 50 NAFLD patients by Chang et al. (2020) demonstrated that acylcarnitine accumulation and disturbance were associated with NAFLD. Serum even-numbered-carbon long-chain acylcarnitines may be a potential biomarker for early clinical screening for NAFLD.

Flores et al. (2021) analyzed the serum lipid profile of 98 Mexican NAFLD patients using direct infusion-tandem mass spectrometry (DI-MS/MS), which found significant differences in serum triacylglycerols, LPC, cholesteryl esters and sphingomyelin in NAFLD patients and indicated that evaluating triacylglycerols or specific lipid combinations may be useful clinical tools for diagnosing NAFLD.

Zhou et al. (2016) found that differential metabolites (glutamate, isoleucine, glycine, LPC, phosphoethanolamine, and aspartate aminotransferase (AST), fasting insulin, and patatin-like phospholipase domain-containing (PNPLA3) genotype were evidently better than those according to clinical or metabolic characteristics separately.

Liu L. et al. (2021) used ultra-performance liquid chromatography-quadrupole time-of-flight tandem mass spectrometry (UPLC-Q/TOF-MS) to analyze serum metabolites in NAFLD patients, and found 5 potential biomarkers including arachidonic acid, pregnenolone sulfate, indoxylsulfuric acid, prasterone, and sebacic acid, which can form a diagnostic model for screening NAFLD. It was also found that there were differences in the metabolism of amino acids and fatty acids in obese and lean NAFLD patients.

Koch et al. (2017) used an untargeted metabolomics to measure serum samples from 555 patients with fatty liver in northern Germany, using magnetic resonance imaging to quantify the fat content in the liver as signal intensity for BCAAs and derived γ-glutamyl dipeptides. Compositional metabolomics scores were correlated with liver signal intensity and FLD. Thus, serum metabolomics signatures were correlated with FLD and the fat content in the liver.

### Metabolomics characterization of NAFLD in basic research

Basic research is essential to clarify the pathological mechanism of NAFLD and to develop new drug targets (Jahn et al., 2019). In recent years, numerous studies using animal and cellular models of NAFLD have been published (Wang et al., 2018; Zhu. et al., 2019; Lee et al., 2020; Wang et al., 2020; Diniz et al., 2021; Li et al., 2021). Wang et al. (2020) untargeted metabolomics analysis of NASH was performed using UPLC-Q/TOF-MS. It was found that sphingomyelin, oleic acid and phosphatidylethanolamine in NASH mice levels were elevated substantially and adenosine levels were reduced significantly. In the development of NASH, glycerophospholipid metabolism is clearly affected. High levels of sphingomyelin may be associated with the expression of acid sphingomyelinase. These altered metabolites may be potential molecular biomarkers for NASH.

Ye et al. (2018) performed a fecal metabolomics analysis of the methionine and choline deficient (MCD)-induced NASH mouse model and found that at 2 weeks, arachidonic acid, hexadecane, palmitic acid, and tetracosane were distinctly different in the control group, and at 4 weeks, cholic acid, selected cholesterol, arachidonic acid, tetracosane, and stearic acid, which provide new possibilities for early diagnosis of NASH.

Yan et al. (2022) used GC-MS targeted metabolic analysis to analyze 36 medium and long-chain fatty acids in the serum of HFD diet-induced mice and found that arachidonic acid, palmitic acid, oleic acid, and stearic acid in the serum of NAFLD mice were significantly elevated. Therefore, these fatty acids can be used as potential biomarkers for the diagnosis of NAFLD. Rom et al. (2020) found that impaired glycine metabolism was closely related to the pathogenesis of NAFLD. They applied metagenomic, transcriptomic, and metabolomics analysis of mouse models and found that glycine-based therapy stimulated liver fatty acid by stimulating liver fatty acid β-Oxidation and glutathione synthesis to slow the development of NAFLD in experimental mice, which provides a new possibility for the treatment of NAFLD.

### Metabolomics in different stages of NAFLD

Metabolomic characterization of NAFLD patients may be a valuable tool for non-invasively distinguishing different stages of NAFLD (Dong et al., 2017; Masarone et al., 2021). Masarone et al. (2021) included 2 study cohorts for metabolomics analysis of serum from NAFLD patients. The first cohort was derived from 69 healthy controls and 144 patients (including 78 with steatosis, 23 with NASH, 15 with NASH cirrhosis, 8 with hepatitis C virus (HCV) cirrhosis and 20 with cryptogenic cirrhosis). The second validation cohort included 44 healthy controls and 50 patients (34 with steatosis, 10 with NASH and 6 with NASH cirrhosis). It was found that taurocholic acid, glycocholic acid, phenylalanine, BCAAs increased with the severity of the disease, from steatosis to NASH, NASH cirrhosis, and decreased with Glutathione. Furthermore, a machine learning model (including 10 different models) was built to validate the diagnostic capability, with >80% accuracy in NAFLD clinical stage prediction. This study suggests that metabolomics signatures of NAFLD patients can serve as a useful method to non-intrusively diagnose NAFLD and differentiate between different stages of the illness, leading to gain an in-depth understanding of its pathology.

Ioannou et al. (2020) analyzed fasting serum samples with nuclear magnetic resonance (NMR) and LC-MS spectroscopy from 57 NAFLD patients, include in 12 with NAFL, 31 with early NASH and 14 with advanced NASH. Spermidine levels were found to correlate with histological severity and with significant decreases in spermidine levels in advanced NASH vs. NAFL, in advanced NASH vs. early NASH and in advanced fibrosis vs. early fibrosis, this indicates that spermidine has a protective against on the progress from NASH to fibrosis. These differences afford mechanistic insights and potentially meaningful metabolic biomarkers that can non-intrusively differentiate patients with NAFL, early NASH, and advanced NASH.

Qi et al. (2017) used high performance liquid chromatography-mass spectrometry (HPLC-MS) to analyze the serum metabolic profiles from NAFLD patients. Add up to 56 metabolites were able to distinguish NASH from NAFL, among which the pyroglutamate was found to be the most promising biomarker. The best critical value was 4.82 mmol/L, and the sensitivity and specificity for the diagnosis of NASH were 72% and 85% respectively. Compared with NAFL patients, the area under the receptor operating characteristic (AUROC) of pyroglutamate levels in NASH was larger than that of adiponectin, interleukin-8 and tumor necrosis factor alpha. This suggests that serum pyroglutamate perhaps is a valuable biomarker for the diagonosis of NASH.

Marques et al. (2021) used liquid chromatography-time-of-flight-mass spectrometry (LC-TOF-MS) to analyze serum samples from patients with NAFL and NASH, which showed that serum leptin levels were proportional to hepatic lipid accumulation and that leptin can be used as a potential biomarker for non-invasive diagnosis of NAFLD. Adiponectin combined with nine serum lipids including PC, TG, and sphingomyelin clearly differentiated NAFL from NASH patients. Ferritin, insulin-like growth factor 1 (IGF-1) and international normalized ratio (INR) may be important factors in the identification of advanced fibrosis.

Kordy et al. (2021) used ultra-high-performance liquid chromatography/tandem mass spectrometry (UHPLC-MS/MS) to analyze the plasma and fecal metabolic profiles of children with NAFL and NASH and found that behenoyl dihydrosphingomyelin can be used to differentiate NASH from NAFL. Glycosyl N-stearoyl-sphingosine and N-stearoyl-sphingosine were important predictors of NASH fibrosis.

Mazzini et al. (2021) used a targeted metabolomics approach for the first time to analyze the plasma and fecal metabolic profiles of metabolic dysfunction-associated fatty liver disease (MAFLD) patients and healthy individuals in a Latin American population, which found significant differences in 24 metabolites. Plasma PC aa C24:0 and PC ae C40:1 were biomarkers that differentiate NAFL from NASH. The PNPLA3 gene was associated with higher level of fatty acid (20:1) and may be a potential metabolic pathway affecting the pathogenesis of MAFLD.

De Mello et al. (2021) analyzed serum metabolites in 233 subjects undergoing bariatric surgery by applying an untargeted metabolomics approach. 164 participants were classified as normal liver, NAFL, or NASH according to the liver histology results. Compared with the normal phenotype, NASH patients had higher levels of fasting serum metabolites, which include tryptophan, phenylalanine and tyrosine, BCAAs (leucine and isoleucine), PC (16:0/16:1) and uridine. Compared with the NAFL group, only tryptophan was increased in the NASH group. Therefore, the differences in serum amino acid metabolism may become an important indicator for non-invasive clinical diagnosis of NASH.

Caussy et al. (2019a) divided patients with biopsy-proven NAFLD into patients without advanced fibrosis and patients with advanced fibrosis, and a combination of 10 serum metabolites was determined by untargeted serum metabolomics analysis. The diagnostic accuracy was higher than the Fibrosis-4 index on the diagnosis of the presence of advanced fibrosis. This study presents that a non-invasive diagnostic test based on blood from NAFLD patients can provide an excellent performance profile for the detection of advanced fibrosis.

Ajaz et al. (2021) evaluated 30 subjects in 3 groups: NAFLD with biopsy-proven mild fibrosis, NAFLD with severe fibrosis, and healthy controls. A metabolomics full-spectrum analysis found that contrast mild/moderate and severe liver fibrosis in NAFLD patients, 14 of 493 quantified metabolites were distinctly changed. Most of the regulated amino acids were components of the urea cycle, including the citrulline/ornithine ratio, arginine, and glutamate. The relevant differential metabolites were used to distinguish the model of patients of NAFLD with mild to moderate fibrosis from patients of NAFLD with severe fibrosis, the AUROC curve was 0.95, the sensitivity was 100%, and the specificity was 80%.

Moolla et al. (2020) analyzed the urinary steroid metabolome of 275 subjects, using GC-MS analysis and machine learning-based generalized matrix learning vector quantification for differentiation of early and advanced fibrosis, NAFLD-related fibrosis and NAFLD-related cirrhosis, NAFLD-related cirrhosis and alcohol-related cirrhosis.

Lewinska et al. (2021) used UPLC-MS to analyze 1,295 metabolites in serum from 249 patients. They found that serum lipid metabolism was rearranged in NAFLD-hepatocellular carcinoma (HCC) and clearly differentiated from noncancerous individuals and other HCC patients. During NAFLD-HCC transformation, unsaturated fatty acids and acylcarnitines (ACs) were gradually reduced, which showed that the increase of fatty acid transporters in NAFLD-HCC tumors resulted in depletion of serum fatty acids.

### Metabolomics in NAFLD with extrahepatic complications

NAFLD is a multisystem disease that not only damages the liver, but also increases the risk of cardiovascular disease (CVD), type 2 diabetes mellitus (T2DM), and chronic kidney disease (CKD) (Byrne and Targher, 2015; Chacko and Reinus, 2016; Adams et al., 2017). More and more studies have begun to explore the interaction between NAFLD and extrahepatic complications, and the impact of NAFLD combined with extrahepatic complications on metabolic function (Younossi et al., 2019; Lee et al., 2021; Sun D.-Q. et al., 2021). Tiwari-Heckler et al. (2018) used LC-MS/MS to measure serum phospholipids in 69 patients divided into NASH and NAFL subgroups, and 28 healthy controls. Compared with healthy controls, the total serum level of PC and sphingomyelin in NAFL and NASH patients was significantly increased. Furthermore, serum lysophosphatidylethanolamine (LPE) levels were distinctly reduced in patients with NAFL and NASH. Circulating PC species, containing α-linoleic acids and linolenic were significantly elevated in NAFLD patients with high blood pressure in comparison with those without high blood pressure. There were no significant differences of phospholipid patterns between NAFLD patients with and without high blood glucose. Whereas, patients with NAFLD who had high blood glucose had significantly higher monounsaturated phosphatidylethanolamine levels than those with lower blood glucose levels.

Martínez-Arranz et al. (2022) linked metabolomics signatures to CVD and genetic risk factors, analyzing 1,154 patients with biopsy-proven NAFLD and four mouse models of NAFLD with impaired very-low-density lipoprotein (VLDL)-TG secretion. Serum metabolome analysis of a NAFLD mouse model with impaired VLDL-TG secretion and a NAFLD mouse model with normal VLDL-TG secretion was performed. They identified three metabolic subtypes. Subtype A replicated the metabolome of mice with damaged VLDL-TG secretion. Subtype B showed intermediate signals. Subtype C replicated the metabolome of mice with normal VLDL-TG secretion. While these characteristics were consistent with known CVD and hereditary risk factors, subtype A had a significantly lower risk of CVD. This could explain differences in hepatic and cardiovascular outcomes, providing the clinically stratification with relevant risk.

## Application of metabolomics in the treatment of NAFLD

### Metabolomics in lifestyle interventions for NAFLD

It is currently recognized that poor lifestyle changes, including healthy diet and increased exercise, are the preferred approach for the treatment of early-stage NAFLD (Mazzotti et al., 2018; Bischoff et al., 2022; Mascaró et al., 2022). Babu et al. (2022) used an untargeted LC-MS-based metabolomics analysis method to perform exercise intervention (without dietary changes) on adipose tissue, plasma, urine and feces in four different sample types, metabolic changes associated with NAFLD were determined. The intervention group underwent 12 weeks of high-intensity interval training (HIIT), while the control group maintained a sedentary lifestyle. HIIT significantly reduced fasting blood glucose concentrations and waist circumference; increased maximal oxygen consumption rate and maximal completed load. Metabolite changes in NAFLD patients after HIIT intervention, including increased amino acid levels in adipose tissue and plasma, while decreased levels in urine and feces. In addition, LPE 18:0, LPE 16:0 and other several glycerophospholipids were increased in adipose tissue and plasma, and some lipids were decreased in feces. It shows that HIIT can improve NAFLD status by regulating the glucose, amino acids and lipids metabolism.

Maciejewska et al. (2018) used GC-MS to analyze the fatty acid levels in plasma erythrocyte membranes of 55 NAFLD patients before and after 6 months of healthy diet intervention, and found that after 6 months of dietary intervention, the NAFLD patients’ plasma stearic acids, linoleic acid, palmitoleic acid, arachidonic acid and oleic acid were significantly decreased, and docosahexaenoic acid levels were increased. Fatty acid profiles are potential biomarkers reflecting pathological changes in NAFLD.

### Metabolomics in drug therapy for NAFLD

Currently, there are no FDA-approved drugs for the treatment of NAFLD in clinical practice. But there are already a few drugs in the development process that are showing promising results (Chalasani et al., 2018; Lee et al., 2019; Amerikanou et al., 2021). A controlled study by Ding et al. (2022) showed that compared with treatment with phytosterol ester (PSE) or n-3 polyunsaturated fatty acids (PUFA) alone ratio, combined supplementation with PSE and PUFA (eicosapentaenoic acid + docosahexaenoic acid) was more valid in improving hepatic steatosis. Changes in serum metabolic profiles of NAFLD subjects in response to n-3 PUFAs and PSE were further investigated using an untargeted UPLC-Q/TOF-MS analytical technique. The results showed that the combined supplementation of PSE and n-3 PUFAs obviously improved serum cotent of PC, LPC, perillyl alcohol, and retinol in NAFLD patients after 3 months of intervention. Polyunsaturated fatty acid levels of flavonoids, as well as PC levels (14:0/20:5, 15:0/20:5), LPC (20:5, 22:6) and retinyl esters were associated with the degree of hepatic steatosis negative correlation.

Amlodipine, a calcium channel blocker, is a commonly used antihypertensive drug in clinical practice (Huang et al., 2019; Gao et al., 2021). In recent years, studies have shown that amlodipine also has liver protection and anti-inflammatory effects (Liu et al., 2019; Qasim et al., 2020). Li et al. (2022) used UHPLC-Orbitrap MS to analyze the effect of amlodipine on the metabolic profile of caecal contents in NAFLD-hypertensive mice. The results suggest that amlodipine improves NAFLD symptoms through altered gut microbiota composition, affecting taurine and hypotaurine metabolism.

Liraglutide is a glucagon-like peptide-1 (GLP-1) analog that has been shown in many studies to have antisteatogenic and anti-inflammatory effects (Armstrong et al., 2016; Moreira et al., 2018; Yu et al., 2019; Liu Q. et al., 2020). Elafibranor is a peroxisome proliferator-activated receptor (PPAR)-α/δ dual agonist that is hepatoprotective, improves lipid metabolism and insulin sensitivity (Ratziu et al., 2016; Boeckmans et al., 2019; Westerouen Van Meeteren et al., 2020; Malik et al., 2021). Perakakis et al. (2021) used a metabolomics analysis method to evaluate the effects of liraglutide and elafibranor on the liver lipidome and metabolome of NASH mice, demonstrating that elafibranor can reduce hepatic glycerides, increase phospholipid metabolites, and beneficially modulateoxidative stress, inflammation and fatty acid oxidation. Liraglutide is able to promote bile acid and carbohydrate metabolism, thereby improving liver status in NASH.

### Metabolomics in gut microbiome for NAFLD

The pathogenesis of NAFLD is very complex and is related to both genetic and environmental factors. Studies have demonstrated that the gut microbiota is involved in NAFLD disease progression with complex roles (Brandl and Schnabl, 2017). Yu et al. (2021) used GC-MS, LC-MS and other metabolomics analysis methods to detect the cecal metabolic profile of NAFLD mice and the fecal metabolic profile of NAFLD patients. They found that compared with normal controls, NAFLD patients and NAFLD mice had intestinal metabolites and microbial communities were significantly changed. The main altered metabolites included tryptophan, bile acids and short-chain fatty acids, which were recovered to a certain extent by the supplementation of lactobacillus lactis and pentococcus.

Using liquid chromatography/electrospray ionization-tandem mass spectrometry (LC-ESI-MS/MS) analysis, Jian et al. (2022) found that rifaximin could improve NASH symptoms by reducing deoxycholic acid content by altering the intestinal firmicutes phylum of NASH mice.

### Application of metabolomics in the treatment of NAFLD with traditional Chinese medicine

**FIGURE 1:**
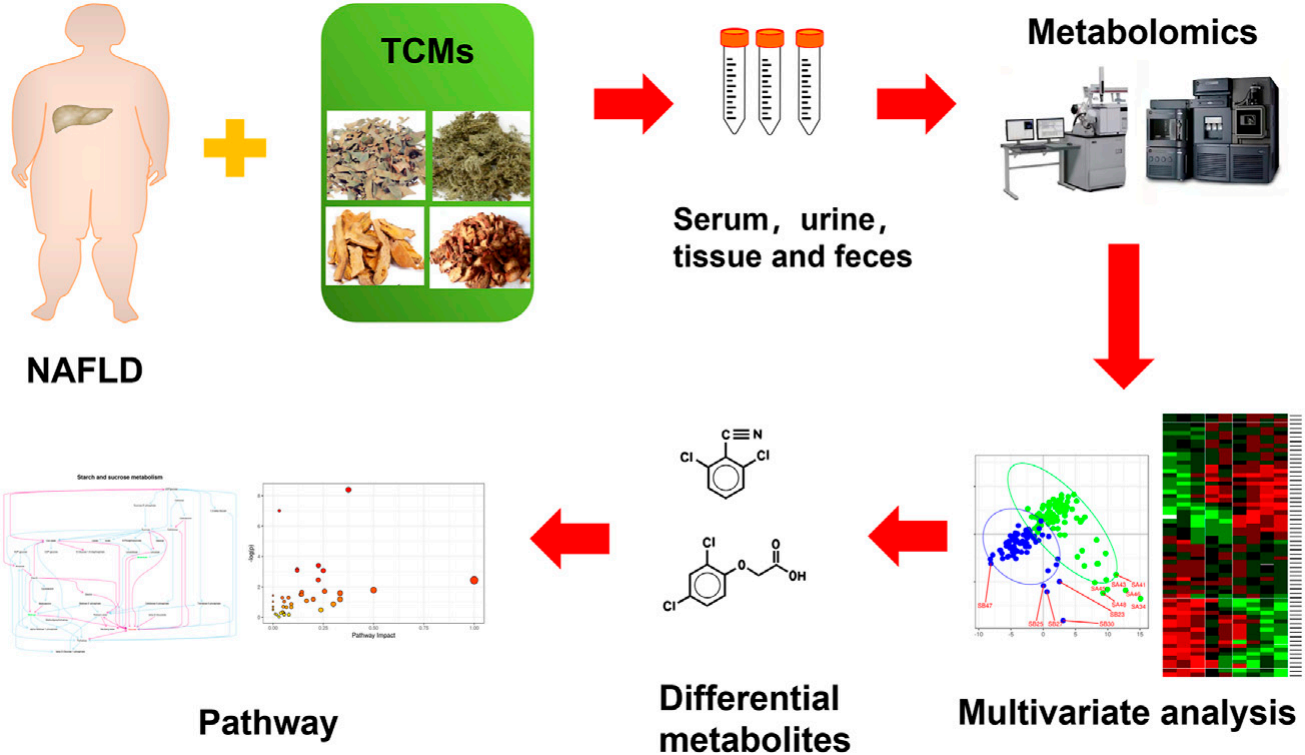
Workflows of metabolomics of TCMs in treatment of NAFLD.

Up to now, there is no clear and effective recommended drug for the treatment of NAFLD at home and abroad. It is generally treated by taking drugs with liver-protecting and anti-inflammatory effects, but the effect is limited and there are many side effects. In mild cases, the digestive tract may be ignored, and in severe cases, it may increase the burden on the liver, resulting in abnormal serum transaminases and lower extremity edema. The incidence of NAFLD is often not caused by a single etiology, but a variety of etiologies affect each other. It is difficult for Western medicine to achieve good results in the treatment of NAFLD, while the compatibility of TCM emphasizes the overall concept and pays attention to syndrome differentiation and treatment. There is no fatty liver disease name in TCM. In Chinese medicine, words such as “liver distension,” “phlegm-drinking” and “flank pain” are closely related to the disease location and main symptoms of NAFLD. The liver is the hub of the body’s metabolism. The three major metabolic carbohydrates, proteins, and amino acids are all carried out in the liver. The liver undertakes most of the metabolic processes of synthesis, decomposition, transformation, and excretion. It is very important to systematically study liver diseases from the perspective of metabolism. Metabolomics belongs to systems biology and omics, and is a significant tool in the research of TCM. It is a new method to find functional small molecules to evaluate the curative effect of TCM (Chashmniam et al., 2019; Hong et al., 2020). Figure 1 shows the metabolomics workflow of TCM for the treatment of NAFLD.

### Classification of traditional Chinese medicine syndrome

In terms of syndrome research, a study conducted by Ma et al. (2017) using TCM syndrome metabolomics method showed that NAFLD patients with damp-heat internal syndrome and liver stagnation and spleen deficiency syndrome had 11 metabolites such as amino acids, lipids, and carbohydrates. The differences indicate that the material basis of different syndromes is different.

Li et al. (2019) found 26 metabolites in the urine of NAFLD rats with liver stagnation and spleen deficiency syndrome, among which 12 metabolites such as creatinine and acetic acid were different between the liver stagnation and spleen deficiency syndrome and the non-liver stagnation and spleen deficiency syndrome.

### Traditional Chinese herbal medicine extract

Gallic acid is a polyphenolic organic compound widely found in rhubarb, cornus officinalis and other plants. Chao et al. (2020) measured gallic acid in a combined mouse model of NAFLD induced by high-fat diet (HFD) and low-dose streptozotocin (STZ)-induced hyperglycemia. Gallic acid in a model that mimics the pathological conditions associated with NAFLD and T2DM. HFD and STZ were found to induce severe diabetes, NAFLD and other metabolic disorders in mice. Daily administration of gallic acid alleviated high blood sugar levels in mice and slowed the progression of NAFLD. The metabolomics results indicated that the hepatoprotective effect of gallic acid on NAFLD combined with T2DM mice was partly achieved by alleviating the disturbance of serum cholesterol metabolism and reducing the oxidative stress in the mice.

Schisandra chinensis caulis polysaccharide (SCP) is the active ingredient in TCM schisandra chinensis, and studies have shown that it has anti-inflammatory, anti-oxidant, lipid-lowering and liver-protecting effects (Huang et al., 2021). Feng et al. (2021) used ultra-high-performance liquid chromatography-quadrupole/electrostatic field orbitrap high-resolution mass spectrometry (UHPLC-Q/Orbitrap-MS/MS) metabolomics method to analyze small molecule metabolites in NAFLD rat blood. The results showed that SCP could upregulate the contents of glucuronic acid, niacin and butyric acid by downregulating the contents of acetylphosphate, fumaric acid, succinic acid, α-ketopentadic acid, proline, sabouramide and citric acid, thereby regulating the expression of related enzymes in the liver of NAFLD rats to alleviate the development of NAFLD.

Silybin refers to the flavonoid lignan compound extracted from the Compositae plant herba silybi, and is widely used as a hepatoprotectant medicine (Federico et al., 2017; Abenavoli et al., 2018). Sun et al. (2020) aimed to explore the metabolic changes regulating by silybin on NAFLD. Experimental mice were fed a high-fat/high-cholesterol (HFHC) diet for 2 months and were given silybin and taurourodeoxycholate for the last 1 month. Serum and liver GC/MS-based metabolomics analysis showed that HFHC diet resulted in abnormal metabolism of metabolites for instance lipid metabolism, tricarboxylic acid (TCA) cycle, amino acid metabolism, polyol metabolism and urea cycle. Both taurourodeoxycholate and silibinin treatment ameliorated the metabolic disorder caused by NAFLD mice.

Berberine is an alkaloid isolated from TCM coptis chinensis, which can improve insulin resistance, lower blood lipids, protect blood vessels, and protect nerves (Cicero and Baggioni, 2016; Imenshahidi and Hosseinzadeh, 2019; Song et al., 2020). Chang et al. (2016) used LC-MS lipidomics to reveal the therapeutic effect of berberine intervention on serum lipid profiles. Berberine significantly altered serum lipid profiles compared with lifestyle intervention alone. Berberine altered sphingolipid metabolism, including a reduction in serum ceramides. These findings demonstrate that lipidomic approaches can be used to elucidate the complex mechanisms of action of specific drugs and are new tools for exploring the mechanisms of NAFLD progression.

Chicory is a medicinal and edible herb (Perović et al., 2021). The polysaccharide in chicory accounts for about one-fifth. Zhu et al. (2019) performed a GC-MS based metabolomics to investigate the therapeutic effect of chicory polysaccharide intervention on the serum metabolic profile of NAFLD rats. Compared with the model group, chicory polysaccharide improved serum lipid and amino acid metabolism disorders in NAFLD rats.

Turmeric extract is the main active ingredient of the TCM turmeric, which has anti-inflammatory, antioxidant, hepatoprotective and anti-tumor effects (Hosseini and Hosseinzadeh, 2018; Soleimani et al., 2018). Wang et al. (2016) performed serum metabolomics using UHPLC-Q/TOF-MS to elucidate the possible mechanism of NAFLD induced by HFD and the therapeutic effect of turmeric extract. The result showed that total turmeric extract has a strong combination of lipid metabolism by affecting steroid hormone biosynthesis, glycerolipid metabolism and glycerophospholipid metabolism signaling pathways effect. While the effects of glucocorticoids on adipose tissue metabolism, and on lipid metabolism in NAFLD rats are controversial, and it should be further studied in the future.

Nuciferine, an active component derived from lotus leaf, has anti-inflammatory, hypolipidemic, hypoglycemic and antitumor effects (Wang et al., 2020a; Wan et al., 2022). Cui et al. (2020a) studies have shown that nuciferine can improve NAFLD in rats by regulating the gene expression of glycerophospholipid, linoleic acid, α-linolenic acid, arginine, proline metabolic pathways and related enzymes.

Puerarin is an active isoflavone glycoside extracted from the TCM Pueraria Lobata, which has the effects of improving insulin resistance, anti-inflammatory, antioxidant and vasodilating (Wang et al., 2006; Chen et al., 2018; Xu et al., 2020). Gong et al. (2021) analyzed the effect of puerarin on the liver and urine metabolic phenotypes of NASH mice based on NMR metabolomics, and identified 8 hepatic differential metabolites and 13 urinary differential metabolites associated with NASH. The metabolic pathways involved include the TCA cycle, one-carbon metabolism, glycolysis, amino acid metabolism, synthesis and degradation of ketone bodies, and pyrimidine metabolism. Puerarin ameliorates hepatic steatosis and inflammation in NASH mice by modulating Helicobacter and Roseburia.

Hyperoside is a flavonoid compound mainly found in plants such as forsythia, hawthorn and dodder, which has many biological effects, such as anti-inflammatory, antioxidant, liver protection and anticancer effects (Sun B. et al., 2021; Wang et al., 2022). Wang et al. (2021) study found that hyperoside can promote the β-oxidation of free fatty acids by activating the expression of farnesoid X receptor (FXR) in the liver of NAFLD rats, thereby reducing lipogenesis, increasing cholesterol efflux and BAs excretion, and achieving the effect of treating NAFLD.

Table 1 lists some other biomarkers and differential metabolic pathways found in NAFLD by metabolomics. TCM has many regulatory effects on these metabolites and metabolic pathways. These findings provide a basis for further understanding of the pathogenesis of NAFLD and help to improve the diagnosis and treatment of NAFLD patients.

**TABLE 1:** The therapeutic effect of Chinese medicine extract on NAFLD.

Method	Monomers/Extracts	Object	Model	Major findings	Ref.
NMR	Gallic acid	Mice	NASH + T2DM	Blocks metabolic disturbance pathways associated with glucose, lipids, amino acids, purines and pyrimidines.	Chao et al. (2020)
UHPLC-Q/Orbitrap-MS/MS	SCP	Rat	NAFLD	Upregulated glucuronic acid, niacin and butyric acid, downregulated acetylphosphate, fumaric acid, succinic acid, α-ketopentadic acid, proline, sabouramide and citric acid.	Feng et al. (2021)
GC-MS	Silybin	Mice	NAFLD	Various metabolic disorders were reversed.	Sun et al. (2020)
LC-MS	Berberine	Human	NAFLD	Decreases serum ceramides and alters sphingolipid metabolism.	Chang et al. (2016)
GC-MS	Chicory polysaccharide	Rat	NAFLD	Improve blood lipid and amino acid metabolism disorders.	Zhu H. et al. (2019)
UHPLC-Q/TOF-MS	Turmeric extract	Rat	NAFLD	Regulates glycerophospholipid metabolism may be involved in the therapeutic.	Wang et al. (2020b)
LC-MS	Nuciferine	Rat	NAFLD	Regulates the gene expression of glycerophospholipid, linoleic acid, α-linolenic acid, arginine, proline metabolic pathways and related enzymes.	Cui et al. (2020a)
NMR	Puerarin	Mice	NASH	Significantly reversed all potential biomarkers associated with NASH in mice.	Gong M.-J. et al. (2021)
LC-MS	Hyperoside	Rat	NAFLD	Reduced hepatic total cholesterol (TC) and TG levels, and enhanced the expression of bile secretion pathway, cholesterol metabolism pathway, fatty acid degradation pathway and FXR.	Wang et al. (2021)

### Traditional Chinese herbal formulas

Traditional Chinese herbal formulas, composed of single Chinese herbal medicines with pharmacological and pharmacodynamic compatibility, are the main form of Chinese medicine. When single Chinese herbal medicines are combined into traditional Chinese herbal formulae, it is different from the original effect of a single drug and can be better applicated in more diseases with complex symptoms. Traditional Chinese herbal formulas represent a complex system with multiple components, targets and effects (Cai et al., 2018). Due to the rich experience in clinical application in the prevention and treatment of NAFLD of Chinese herbal formulae, it is attracting increasingly attention from researchers around the world. Metabolomics analysis has been applied widely for the pharmacology of liver-related disease and therapies (Table 2).

**TABLE 2:** The therapeutic effect and mechanisms of Chinese herbal formulas for NAFLD.

TCM	Method	Object	Model	Mechanism	Ref.
YCHD	UPLC-Q/TOF-MS	Rar	NAFLD	Regulates glycerophospholipid metabolism, α-linolenic acid metabolism, linoleic acid metabolism and nicotine and nicotine amine metabolism.	Deng et al. (2019)
SLBZS	UHPLC-MS	Rat	NAFLD	Increased the levels of some sphingolipids, including ceramide (d18:1/23:0) and sphingomyelin (d16:1/18:0).	Deng et al. (2019)
DCHD	LC–MS	Rat	NAFLD	Reversed the disorder of pentose phosphate, glycine/serine/threonine, arachidonic acid and glycerophospholipid metabolic pathways.	Cui et al. (2020b)
THSWT	UPLC/Q-TOF-MS	Mice	NASH	Reversed the increased expression of key enzymes in glycerophospholipid metabolism (lysophospholipase 3 and neuropathy target esterase).	Park et al. (2020)
QGE	GC-MS	Mice	NASH	Decreased liver and serum bile acid concentrations and increased fecal lithocholic acid. Increased lithocholic acid-producing bacteroides and clostridium.	Li et al. (2020)

Wang et al. (2020b) applied UPLC-Q/TOF-MS technology found that Yin-Chen-Hao Decoction (YCHD) can treat NAFLD by regulating linoleic acid metabolism, glycerophospholipid metabolism, α-linolenic acid metabolism, and nicotine and nicotine amine metabolism.

Shen-Ling-Bai-Zhu-San (SLBZS), is a classic traditional Chinese herbal formulae. Deng et al. (2019) used the untargeted lipidomics method of UHPLC-MS to study the effect of SLBZS on the liver lipid profile of NAFLD rats. HFD feeding significantly altered several ceramide and sphingomyelin species, and SLBZS administration increased the levels of some sphingolipids, including ceramide and sphingomyelin. This finding suggests SLBZS improved NAFLD through regulating sphingolipid metabolism, and related enzymes related to the sphingolipid synthesis possible critical goals for the treatment of NAFLD.

Da-Chai-Hu Decoction (DCHD) is a classic prescription for treating liver-stomach heat pattern from the Treatise on Febrile and Miscellaneous Diseases (Ren et al., 2020). Cui et al. (2020b) used non-targeted metabolomics to study the changes of metabolites in serum of NAFLD rats and concluded that DCHD treatment improved the disorder of glycine/serine/threonine, pentose phosphate, arachidonic acid and glycerophospholipid metabolic pathways in NAFLD model.

Tao-Hong-Si-Wu-Tang (THSWT) is a traditional herbal formula with the roles of promoting the blood circulation and inhibiting inflammation. Park et al. (2020) used the UPLC-Q/TOF MS method to analyze the effects of THSWT on lipid metabolism patterns in HFHC diet mice. The results showed that THSWT may ameliorate liver injury in NASH mice by modulating lipid metabolism, such as phospholipids and sphingolipids. THSWT significantly reversed the increased expression of key enzymes in glycerophospholipid metabolism (lysophospholipase 3 and neuropathy target esterase). This proves that lipidomic analysis is helpful to study the therapeutic effect of TCM herbal decoction.

Qiang-Gan Formula is a traditional Chinese herbal formulae containing 16 kinds of TCMs. Clinical studies have proved that its liver-protecting effect is obvious, but the specific mechanism is still unclear (Zhu M. et al., 2019). Li et al. (2020) analyzed bile acid profiles in serum, liver, and fecal samples of NASH mice by GC-MS technology, and detected the fecal microbiota. The results suggest that Qiang-Gan Formula extract (QGE) improved liver inflammation, decreased liver and serum bile acid concentrations, and increased fecal lithocholic acid in NASH mice. In addition, QGE increased the lithocholic acid-producing species bacteroides and clostridium in NASH mice, thereby altering the structure of the gut microbiota.

## Challenge and perspectives

Metabolomics technology has been widely used in clinical and basic research to reflect the affected metabolic pathways by detecting the content of metabolites in living organisms. It has great value in disease diagnosis, pathological mechanism, and drug efficacy evaluation. However, there are still some common problems in the current international metabolomics research. For example, there are huge differences in the study sample size of different countries and different ethnic groups. There are also errors in the detection of different samples and different instrument platforms. The methods for analyzing and extracting metabolites on various experimental platforms are not uniform. These issues limit further discoveries from metabolomics studies. The solutions include expanding the sample size, collecting samples from multiple regions and multiple centers, unifying quantitative methods, unifying quality control standards and mixing standards. Most of the current researches are non-targeted metabolomics studies, which stay at the level of discovering differential metabolites, and rely too much on the literature for the biological explanation of differential metabolites. The research on the mechanism is not deep enough, and we should focus on some specific metabolites and their mechanisms of action for in-depth research. The study of the metabolic characteristics of gut microbes is a promising strategy for the prevention and treatment of NAFLD, but the non-replicability of gut microbiota is a major difficulty hindering in-depth clinical research.

Metabolomics helps to understand the metabolic changes of a whole under different physiological and pathological conditions, and this characteristic is very consistent with the holistic concept of TCM. Therefore, metabolomics has become an important method to support modern TCM research. However, there are still some problems in the research of TCM with metabolomics technology, which need to be solved. For example, in basic research, choosing different animal models and different modeling methods will have an impact on the transformation of metabolites. The same traditional Chinese herbal formulas or monomers may also cause different metabolite regulation due to the selection of different medicinal parts of medicinal materials, different compatibility ratios, and different processing methods, thus affecting the screening of metabolites and metabolic pathways. Quality control of TCM is the basis for clinical efficacy, and metabolomics analysis methods are used to analyze the key factors affecting the quality of TCM materials, and to determine the factors related to quality changes, promote the standardization of the quality control system of TCM, thereby improving the efficacy and safety of TCM. In addition, metabolomics is integrated with genomics, transcriptomics, proteomics, network pharmacology and clinical biochemistry, etc., enables comprehensive and systematic research.

There is a compatibility relationship in TCM compounds, and the chemical components contained are complex, with the characteristics of multi-component, multi-level, multi-target and multi-metabolic pathways. The TCM compound and monomer control metabolites have their own merits, and the emphasis is different. Compared with traditional Chinese herbal formulas, the monomer components and therapeutic effects of TCM are relatively simple, so we can focus on some specific metabolites and their mechanism of action for in-depth research. Traditional Chinese herbal formulas are a variety of bioactive components that contain synergistic effects. During clinical research, we focus on the efficacy and side effects of drugs. To explore the mechanism of action of traditional Chinese herbal formulas and monomers, and to provide scientific basis for clinical diagnosis, syndrome classification, and pharmacodynamic mechanism, further research is required in the future.

## Conclusion

NAFLD is one of the leading causes of liver cancer mortality and morbidity worldwide. Although many measures have been taken to prevent NAFLD and manage the risk, and new therapies have been adopted to treat existing NAFLD, the quality of life of patients has been greatly affected. Thus, it is important to identify new characteristic factors and therapeutic targets for early diagnosis. Metabolomics offers a new approach to solve this problem, providing a detailed analysis of the metabolic state and helping to gain deep understanding into the molecular mechanisms of NAFLD. Quantifying a large number of circulating metabolites in a variety of different pathways can also determine the metabolic changes before the onset of obvious diseases, making it possible to identify NAFLD risk individuals earlier and more accurately. It can also elucidate the mechanism of TCM on the treatment of NAFLD (Shi et al., 2020; Dai et al., 2021). With the continuous refinement and development of metabolomics, the modernization of TCM will continue to advance, and great progress will be made in the diagnosis, prevention and treatment of NAFLD. The discovery and application of potential biomarkers for different stages of NAFLD will become a reality, vastly improving the diagnosis and treatment level of NAFLD, and improving the prognosis of patients.

## Glossary

ACs: acylcarnitines
AST: aspartate aminotransferase
AUC: area under the curve
AUROC: area under the receptor operating characteristic
BCAAs: branched-chain amino acids
CKD: chronic kidney disease
CVD: cardiovascular disease
DCHD: Da-Chai-Hu Decoction
DI-MS/MS: direct infusion-tandem mass spectrometry
GC-MS: gas chromatography-mass spectrometry
FXR: farnesoid X receptor
HCC: hepatocellular carcinoma
HCV: hepatitis C virus
HFD: high-fat diet
HFHC: high-fat/high-cholesterol diet
HIIT: high-intensity interval training
HPLC-MS: high performance liquid chromatography-mass spectrometry
LC-ESI-MS/MS: liquid chromatography/electrospray ionization-tandem mass spectrometry
LC-MS: liquid chromatography-mass spectrometry
LPC: lysophosphatidylcholine
LPE: lysophosphatidylethanolamine
MAFLD: metabolic dysfunction-associated fatty liver disease
MCD: methionine and choline deficient
NAFL: nonalcoholic simple fatty liver
NAFLD: non-alcoholic fatty liver disease
NASH: non-alcoholic steatohepatitis
NMR: nuclear magnetic resonance
PC: phosphatidylcholine
PNPLA3: patatin-like phospholipase domain-containing protein 3
PSE: phytosterol ester
QGE: Qiang-Gan Formula extract
ROC: receiver operating characteristic
SCP: schisandra chinensis caulis polysaccharides
SLBZS: Shen-Ling-Bai-Zhu-San
STZ: streptozotocin
T2DM: type 2 diabetes mellitus
TC: total cholesterol
TG: triglyceride
TCA: tricarboxylic acid cycle
TCM: traditional Chinese medicine
THSWT: Tao-Hong-Si-Wu-Tang
UHPLC-MS/MS: ultra-high-performance liquid chromatography tandem mass spectrometry
UHPLC-Q/Orbitrap-MS/MS: ultra-high-performance liquid chromatography-quadrupole/electrostatic field orbitrap high-resolution mass spectrometry
UPLC-MS: ultra-performance liquid chromatography mass spectrometry
UPLC-Orbitrap MS: ultra-performance liquid chromatography-orbitrap mass spectrometry
UPLC-Q/TOF-MS: ultra-performance liquid chromatography-quadrupole/time-of-flight mass spectrometry
UPLC-Q/TOF-MS/MS: ultra-performance liquid chromatography-quadrupole/time-of-flight tandem mass spectrometry
VIP: variable importance in projection
VLDL: very low density lipoprotein
